# Atorvastatin Increases the Expression of Long Non-Coding RNAs ARSR and CHROME in Hypercholesterolemic Patients: A Pilot Study

**DOI:** 10.3390/ph13110382

**Published:** 2020-11-12

**Authors:** Isis Paez, Yalena Prado, Carmen G. Ubilla, Tomás Zambrano, Luis A. Salazar

**Affiliations:** 1 Center of Molecular Biology and Pharmacogenetics, Department of Basic Sciences, Faculty of Medicine, Universidad de La Frontera, 4811230 Temuco, Chile; isis.paez921007@gmail.com (I.P.); yalenapradovizcaino@gmail.com (Y.P.); carubilla@yahoo.com (C.G.U.); 2 Department of Medical Technology, Faculty of Medicine, Universidad de Chile, 8380453 Santiago, Chile; tomas.zambrano@uchile.cl

**Keywords:** atorvastatin, epidrugs, lncRNAs, LASER, ARSR, CHROME, hypercholesterolemia

## Abstract

Atorvastatin is extensively used to treat hypercholesterolemia. However, the wide interindividual variability observed in response to this drug still needs further elucidation. Nowadays, the biology of long non-coding RNAs (lncRNAs) is better understood, and some of these molecules have been related to cholesterol metabolism. Therefore, they could provide additional information on variability in response to statins. The objective of this research was to evaluate the effect of atorvastatin on three lncRNAs (lncRNA ARSR: Activated in renal cell carcinoma (RCC) with sunitinib resistance, ENST00000424980; lncRNA LASER: lipid associated single nucleotide polymorphism locus, ENSG00000237937; and lncRNA CHROME: cholesterol homeostasis regulator of miRNA expression, ENSG00000223960) associated with genes involved in cholesterol metabolism as predictors of lipid-lowering therapy performance. Twenty hypercholesterolemic patients were treated for four weeks with atorvastatin (20 mg/day). The lipid profile was determined before and after drug administration using conventional assays. The expression of lncRNAs was assessed in peripheral blood samples by RT-qPCR. As expected, atorvastatin improved the lipid profile, decreasing total cholesterol, LDL-C, and the TC/HDL-C ratio (*p* < 0.0001) while increasing the expression of lncRNAs ARSR and CHROME (*p* < 0.0001) upon completion of treatment. LASER did not show significant differences among the groups (*p* = 0.50). Our results indicate that atorvastatin modulates the expression of cholesterol-related lncRNAs differentially, suggesting that these molecules play a role in the variability of response to this drug; however, additional studies are needed to disclose the implication of this differential regulation on statin response.

## 1. Introduction

Heart diseases continue to be the leading cause of death in developed and developing countries. A significant risk factor prone to be modified in these diseases is represented by dyslipidemia, which corresponds to a disequilibrium in cholesterol homeostasis [1,2]. High cholesterol levels in the blood, particularly low-density lipoprotein cholesterol (LDL-C), have been associated with a higher risk of atherosclerosis, brain stroke, and chronic heart disease [3]. In the last decades, the appropriate control and early treatment of hypercholesterolemia have been an important strategy to reduce mortality and morbidity rates from cardiovascular events [4]. The use of lipid-lowering drugs like HMG-CoA reductase inhibitors (statins) is currently the preferred therapeutic strategy to decrease LDL-C levels [5,6], and nowadays, several statins can be found in the market [7], but atorvastatin is usually the drug of choice because of its potent lipid-lowering effect, partly attributed to its higher lipophilicity, and low IC^50^, among others [8]. However, even though statins are safe and well-tolerated, a by genetic and environmental factors. Among these factors, we can find biologic and substantial proportion of patients undergoing treatment do not achieve proper lipid reductions. Therefore, they are considered refractory to this type of drug, i.e., statin-resistant. Once atorvastatin has been administered, reports show a differential response to the same dose among patients [9,10]. Moreover, we have previously reported a significant variation in response to atorvastatin in Chilean hypercholesteremic individuals [11,12], ranging between 5% and 70% of LDL-C reductions, an outcome partly conditioned physiological conditions (absorption and metabolism), age, gender, race, adherence to treatment, medical prescription, and oral doses [10,13,14,15,16].

LncRNAs are defined as transcripts of long RNAs of more than 200 nucleotides that cannot be translated into proteins [17]. Recently, lncRNAs have been proposed as a novel approach for treating hypercholesterolemia [18]. For example, cholesterol homeostasis regulator of miRNA expression (CHROME, ENSG00000223960), which has been recently identified in primates, has been found elevated in plasma and atherosclerotic plaques of individuals with coronary artery disease (CAD). CHROME expression is influenced by dietary and cellular cholesterol via the sterol-activated liver X receptor transcription factors, which control genes that mediate the response to cholesterol overload. CHROME promotes cholesterol efflux and high-density lipoproteins (HDL) biogenesis, while it stops the action of a group of functionally related miRNAs that repress genes in those pathways. CHROME knockdown in human hepatocytes and macrophages is associated with an increase of microRNAs (miRs) miR-27b, miR-33a, miR-33b, and miR-128 levels [19]. On the other hand, the lncRNA ARSR (Activated in renal cell carcinoma (RCC) with Sunitinib Resistance, ENST00000424980) promotes hepatic cholesterol biosynthesis via Akt/SREBP-2/HMGCR modulation. ARSR overexpression has also been associated with increased HMG-CoA reductase (HMGCR) expression, a key enzyme regulating cholesterol synthesis [20]. Moreover, other lncRNAs have also been involved in lipid metabolism [21,22], such as MALAT1 [23], H19 [24,25], lncHR1 [26], APOA1-AS [27] and APOA4-AS [28], AT102202 [29], lnc-HC [30], HULC [31], lncLSTR [32], RP5.883A20.1 [33], lincRNA-DYNLRB2–2 [34], and MeXis [35].

So far, few reports have studied the role of lncRNAs and statins. Recently, Mitchel and colleagues demonstrated that RP1-13D10.2 plays a role as a novel modulator of changes induced by statins in cholesterol metabolism [36]. On the other hand, lncRNA in lipid-associated single nucleotide polymorphism gene region (LASER, ENSG00000237937), a novel lncRNA, showed a positive correlation with plasma levels of proprotein convertase subtilisin/kexin type 9 (PCSK9) in statin-naïve patients. Following treatment, LASER and PCSK9 expression increased simultaneously in both human and an in vitro model of HepG2 cells [37]. Additionally, MANTIS, a pro-angiogenic long non-coding RNA in vascular disease development, is tightly regulated by the transcription factors KLF2 and KLF4. MANTIS limits ICAM-1 mediated monocyte adhesion to endothelial cells and thus atherosclerosis development in humans. Moreover, MANTIS has also been shown to be relevant to the mediation of atorvastatin’s pleiotropic effects [38].

Considering that lncRNAs are emerging as essential mechanisms involved in lipid metabolism and play intricate roles in controlling transcriptional and post-transcriptional regulatory pathways, lncRNAs represent meaningful candidates as statin predictor responses. Consequently, we evaluated atorvastatin’s effect on three lncRNAs (ARSR, LASER, and CHROME), which have been associated with genes involved in cholesterol metabolism, as predictors of lipid-lowering therapy in Chilean patients with hypercholesterolemia treated with 20 mg of atorvastatin for four weeks.

## 2. Results

### 2.1. Clinical and Demographic Characteristics

Table 1 shows the clinical and demographic characteristics of the individuals evaluated before statin treatment (baseline). Hepatic enzymes were normal after therapy. None of the individuals experienced adverse effects. 

Lipid levels before and after treatment with atorvastatin are shown in Table 2. As it was expected, statin treatment reduced total cholesterol (TC) and LDL-C levels, and TC/HDL-C index (*p* < 0.0001). However, there was no significant effect on HDL-C, VLDL-C, and TG levels (*p* > 0.05).

### 2.2. Atorvastatin Effect on LDLR Gene and lncRNAs Expression in Hypercholesterolemic Patients Following Statin Therapy

**Figure 1:**
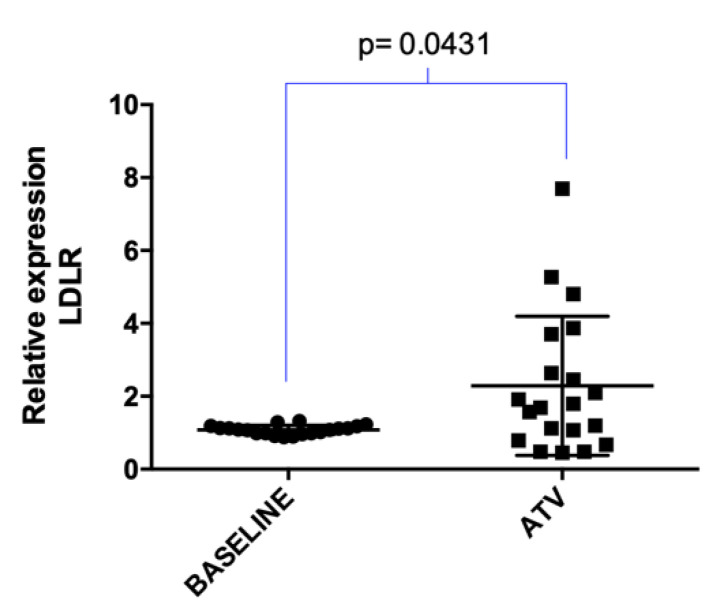
Expression of low-density lipoprotein receptor (*LDLR*) in hypercholesterolemic (HC) patients treated with atorvastatin. Relative quantification was completed by real-time PCR from total RNA extracted from leukocytes from HC patients before (baseline) and after treatment with atorvastatin (ATV, 20 mg/day/4 weeks). *p*-value was obtained by paired *t*-Test. Normalization was done using ribosomal protein L27 (RPL27) as the reference gene.

The treatment with atorvastatin (20 mg/day/4 weeks) positively regulated low-density lipoprotein receptor (*LDLR*) expression in hypercholesterolemic patients (*p* = 0.0431) (Figure 1). 

**Figure 2:**
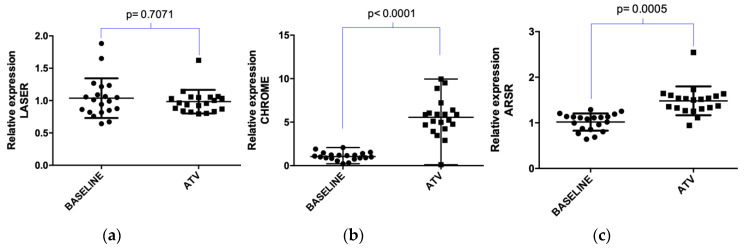
Expression of lncRNAs in hypercholesterolemic (HC) patients treated with atorvastatin: (**a**) LASER expression; (**b**) ARSR expression; and (**c**) CHROME expression. Relative quantification was performed by real-time PCR from total RNA extracted from leukocyte cells of peripheral blood from HC patients before (baseline) and after treatment with atorvastatin (ATV, 20 mg/day/4 weeks). *p*-value was obtained by paired *t*-Test. Normalization was performed using small nuclear RNA U6 (U6) as a reference gene.

Additionally, atorvastatin positively regulated ARSR (*p* = 0.0005) and CHROME (*p* < 0.0001) expression in hypercholesterolemic patients. The lncRNA LASER did not show significant differences once the treatment was completed (*p* = 0.7071) (Figure 2).

## 3. Discussion

LncRNAs play roles in important physiological processes, and their deregulation contributes to the pathogenesis of several diseases. Additionally, several lncRNAs have been implicated in the regulation of cholesterol metabolism. However, in the context of statin treatment, few investigations have focused on the role portrayed by lncRNAs. Thus, we evaluated the impact of 20 mg/day atorvastatin on the expression of LASER, ARSR, and CHROME in hypercholesterolemic patients, observing that atorvastatin effectively optimized the lipid profile, reducing total cholesterol and LDL-C serum levels. The most significant reduction was observed in LDL-C, reaching a 44.6% reduction. However, atorvastatin did not induce changes in HDL-C and TG levels. Literature about this topic shows controversial data because high doses of atorvastatin have been associated with increased HDL-C levels [39]. However, a reverse relationship has also been observed between the increase of the lipids fraction and statin dose [40]. On the other hand, it has been reported that low-dose atorvastatin does not produce changes in the HDL-C concentration in plasma [41]. 

Concerning lncRNA expression, data showed that atorvastatin increased the expression of lncRNAs ARSR and CHROME. Concomitantly, LDLR expression was also elevated in peripheral blood leukocytes, which is consistent with additional reports [42]. However, the lncRNA LASER was unaffected after the treatment. Regarding LASER, reports show that this lncRNA is highly expressed in hepatocytes treated with atorvastatin for 24 h using different doses (1–40 μM) [37]. In the same study, authors demonstrate that LASER increased its expression significantly after therapy with 20 mg/day atorvastatin during five days in CMSP in 11 CAD patients. Furthermore, LASER expression was elevated in peripheral blood mononuclear cells (PBMC) from 26 hypercholesterolemic patients vs. 149 normocholesterolemic individuals [37]. Differences observed between studies may respond to treatment duration and the inclusion/exclusion criteria for patient’s enrollment, among others.

On the other hand, Huang et al. documented increased expression of ARSR in the serum of 30 hypercholesterolemic individuals compared to 20 healthy subjects. Additionally, the authors showed increased lncRNA expression in hepatic cells of mice fed with a cholesterol-rich diet (4%) vs. mice fed with a standard diet in 20 weeks (*n* = 10/group) [20]. A recent study also reported that CHROME increases its expression in plasma on CAD patients (*n* = 14). In the same way, authors reported an increased expression in the liver of African monkeys fed with a fat-rich diet and moderate in cholesterol during eight weeks vs. a basal diet. Similar results were found in HepG2 cells treated with cholesterol-cyclodextrin (10 µg/mL; 72 h) compared to the vehicle, and in human THP-1 macrophages treated with acetylated LDL (acLDL; 37.5 µg/mL; 24 h) compared to untreated cells [19]. 

While we report two upregulated lncRNAs following atorvastatin treatment, several limitations must be noted to interpret our results better. First, our data come from an unbalanced male/female ratio, which accounts for a variable not currently evaluated that could partly contribute to the differences encountered. Second, the small sample size implies that these results must be replicated in a wider population, even though we present conclusions based on a cohort that is sufficient to detect a four-fold difference with at least 90% power. Finally, experimental constraints impede providing a mechanistic insight mainly due to limited gene expression evaluation; however, assays were properly conducted considering international guidelines [43]. Nonetheless, our work shows a positive and significant regulation of CHROME, and to a lesser extent of ARSR, in leukocyte cells of peripheral blood of hypercholesterolemic patients and constitutes a first approach to the role that statins play on lncRNAs regulation; however, additional research is necessary to clarify further the biological impact of these lncRNAs on cholesterol homeostasis and statin treatment.

## 4. Materials and Methods 

### 4.1. Individual’s Selection and Treatment Protocol

A total of 20 (6 men and 14 women) unrelated Chilean individuals (47.30 ± 11.35 years) diagnosed with hypercholesterolemia according to the NCEP criteria [44] were selected from Chol-Chol Familial Health Center (Temuco, La Araucanía, Chile). Patients were treated with 20 mg/day atorvastatin for four weeks. Individuals with familial hypercholesterolemia, hepatic or renal disease, or diabetes mellitus, and patients undergoing medication like diuretics, beta-blockers, concomitant hypolipemiant therapy, and drugs affecting their lipid profile, were excluded. All patients agreed to participate voluntarily by signing a written informed consent. The Scientific Ethics Committee of the Universidad de La Frontera approved this study protocol (N° 045_17), and the investigation was carried out following the ethical principles of the Declaration of Helsinki [45]. 

### 4.2. Biochemical Analysis

Lipid concentrations were determined in blood samples obtained before and after atorvastatin treatment. Samples were collected by a routine venous puncture in vacutainer tubes without anticoagulant after 10–12 h fast. TC, TG, and HDL-C serum levels were performed with standard laboratory methods [46]. The LDL-C fraction was calculated using the Friedewald equation when triglycerides did not exceed 400 mg/dL [47].

### 4.3. Molecular Analysis

EDTA-anticoagulated blood sample was obtained for total RNA isolation of peripheral blood leucocytes. The extraction of total RNA was performed using the mirVana^TM^ kit (Ambion, Applied Biosystems, Houston, TX, USA), according to the manufacturer’s instructions. RNA integrity and quantification were performed using an Infinite^®^ 200 PRO NanoQuant (Tecan Group Ltd., Männedorf, Switzerland). According to the manufacturer’s instructions, cDNA synthesis was carried out with 1 µg of total RNA using the High-Capacity RNA-to-cDNA™ kit (Applied Biosystems, Foster City, CA, USA). Reverse transcription procedure was done according to the manufacturer’s protocol and performed in two steps using a conventional thermal cycler: 37 °C for 60 min. and a final phase of 95 °C for 5 min. The lncRNAs (LASER, ARSR, and CHROME) and LDLR quantification was performed with Fast^®^ SYBR Green Master Mix (Applied Biosystems, Foster City, CA, USA) using real-time PCR, before and after atorvastatin treatment. PCR primer sequences are listed in Table 3. The primers for RPL27 gene expression were designed using Primer-BLAST software. To normalize real-time RT-PCR data, we used the geometric mean of multiple internal controls; for *LDLR*, we tested RPS29, RPL27, and RPS13; and for lncRNAs analyses, we tested small RNA U6, RP11-204K16.1, and XLOC_012542 [48]. Our results show that RPL27 and small RNA U6 were the most stably expressed genes and are optimal reference genes for *LDLR* and lncRNA analysis, respectively. The reactions were subjected to the cycling protocol using the StepOne thermocycler (Applied Biosystems, USA) using the following protocol: initial activation at 95 °C for 20 s, followed by 40 cycles of denaturation at 95 °C for 3 s and one step of annealing/extension at 60 °C for 30 s. Further analyses were carried out using the Threshold Cycle comparative method. 

### 4.4. Statistical Analysis

We used the statistic program GraphPad Prism version 5.0 (GraphPad Software Inc., San Diego, CA, USA). For demographic, clinical, and laboratory variables, we used descriptive statistics. Statistical parameters such as media and standard deviation were used for continuous variables. Lipid levels were analyzed in the basal stage and after atorvastatin treatment; to summarize them, the change percentage was used. Before and after treatment, values were compared with the paired Student’s *t*-Test. Regarding sample size calculation, considering alpha and beta values of 0.05 and 0.2, respectively, the sample size needed to detect a four-fold difference between groups with at least 90% power corresponding to 18 individuals in total. All statistical tests of the hypothesis were two-sided. The level of significance considered was α = 0.05.

## Figures and Tables

**Table 1:** Basal clinical and demographic characteristics of the study group.

Parameter	*n* = 20
Age (years)	47.30 ± 11.35
Men/Women (*n*)	(6/14)
Glycemia (mg/dL)	95.94 ± 6.94
AST/GOT (UI/L)	23.73 ± 5.32
ALT/GPT (UI/L)	30.25 ± 7.14
CK (UI/L)	110.18 ± 25.99
Uremia (mg/dL)	30.88 ± 6.31
Ureic Nitrogen (mg/dL)	13.31 ± 3.18
Blood Creatinin (mg/dL)	1.08 ± 0.16
Hemoglobin (Hb) (g/dL)	13.83 ± 1.15
Hematocrit (Hto.) (%)	41.25 ± 3.04
Leucocytes (×10^3^/μL)	6.68 ± 1.89
Platelets (×10^3^/μL)	243.56 ± 44.06
Total Bilirubin (TB) (mg/dL)	0.52 ± 0.14
Direct Bilirubin (DB) (mg/dL)	0.13 ± 0.04
Indirect Bilirubin (IB) (mg/dL)	0.37 ± 0.14

Values are expressed as mean ± standard deviation. *n*, number of individuals; AST/GOT aspartate aminotransferase; ALT/GPT, alanine aminotransferase; CK, creatine kinase.

**Table 2:** Serum lipid levels at baseline and after treatment with atorvastatin (20 mg/day/4 weeks).

Lipids	Baseline (mg/dL)	Post-Treatment (mg/dL)	Change (%)	*p*-Value
TC	239.35 ± 28.28	158.15 ± 33.41 ***	34.13 ± 10.71	<0.0001
HDL-C	44.45 ± 10.09	41.20 ± 9.48	6.42 ± 14.92	0.3005
LDL-C	164.62 ± 26.32	91.37 ± 28.28 ***	44.61 ± 14.02	<0.0001
VLDL-C	30.78 ± 13.04	24.19 ± 11.23	15.04 ± 36.78	0.0948
TG	150.40 ± 66.23	121.65 ± 55.40	10.96 ± 37.89	0.1447
TC/HDL-C	5.55 ± 0.95	3.92 ± 0.80 ***	28.43 ± 13.33	<0.0001

Results are expressed as mean ± standard deviation. ***: indicates a highly significant *p*-value, which was obtained by paired Student’s *t*-Test. TC, total cholesterol; HDL-C, high-density lipoprotein cholesterol; LDL-C, low-density lipoprotein cholesterol; VLDL-C, very low-density lipoprotein cholesterol; TG, triglycerides; TC/HDL-C, ratio among total cholesterol and high-density lipoprotein cholesterol.

**Table 3:** Sequences of primers used to quantify gene expression by RT-qPCR.

Name	Forward Primer (5’-3’)	Reverse Primer (5’-3’)	Reference
LASER	AAGGTGCCACAGATGCTCAA	GGGAGGTATCCCGGAGAAGT	[37]
ARSR	TTTGAAATGCTCTTTGAGGGAT	TGCAGGTTGTCTGAAGTTGGA	[20]
CHROME	GCAGGAGCTTGAATTTCAGT	TGTACTGAGTGGGCATTTAT	[19]
U6	CTCGCTTCGGCAGCACATATAC	GGAACGCTTCACGAATTTGC	[48]
LDLR	CTGAAATCGCCGTGTTACTG	GCCAATCCCTTGTGACATCT	[49]
RPL27	TCCGGACGCAAAGCTGTCATC	GGTCAATTCCAGCCACCAGAGCAT	-

LASER, lncRNA in lipid-associated single nucleotide polymorphism gene region; ARSR, lncRNA activated in renal cell carcinoma (RCC) with sunitinib resistance; CHROME, cholesterol homeostasis regulator of miRNA expression; U6, small nuclear RNA U6; LDLR, low-density lipoprotein receptor; RPL27, ribosomal protein L27.
